# Few eligible for the newly recommended short course MDR-TB regimen at a large Mumbai private clinic

**DOI:** 10.1186/s12879-019-3726-8

**Published:** 2019-01-28

**Authors:** Zarir F. Udwadia, Jeffrey A. Tornheim, Shashank Ganatra, Andrea DeLuca, Camilla S. Rodrigues, Amita Gupta

**Affiliations:** 1 Medical Research Centre, P.D. Hinduja National Hospital, Veer Savarkar Road, Mahim, Mumbai, Maharashtra 400016 India; 2 Division of Infectious Diseases, Center for Clinical Global Health Education, Johns Hopkins University School of Medicine, 600 North Wolfe Street, Phipps 521, Baltimore, MD 21287 USA; 3 Division of Global Disease Epidemiology and Control, Bloomberg School of Public Health, Johns Hopkins University, Baltimore, MD 21287 USA

**Keywords:** MDR-TB, TB treatment, Bangladesh regimen, Drug susceptibility testing, Short course

## Abstract

**Background:**

India has the world’s highest tuberculosis burden, and Mumbai is particularly affected by multidrug resistant tuberculosis (MDR-TB). WHO recommends short, intensive treatment (“Short Course”) for previously untreated pulmonary MDR-TB patients but does not require universal drug susceptibility testing (DST) before Short Course. DST would likely screen out many MDR-TB patients in places like Mumbai with significant drug resistance.

**Methods:**

MDR-TB patients at a private clinic were recruited for a prospective observational cohort. Short Course eligibility was evaluated by clinical criteria and DST results. Eligibility by DST was classified as rifampin monoresistance (as tested by Xpert MTB/RIF), rifampin, fluoroquinolones, and 2nd-line injectable drugs resistance (as tested by line probe assays) and resistance to other drugs.

**Results:**

Of 559 participants with MDR-TB, 33% met clinical eligibility for Short Course. DST for rifampin, fluoroquinolones, and 2nd-line injectable drugs excluded 74.7% of participants. Complete phenotypic DST excluded 96.6% of participants. Prior treatment with either 1st or 2nd-line drugs did not significantly affect eligibility.

**Conclusions:**

In a global MDR-TB hotspot, < 5% of participants with MDR-TB were appropriate for Short Course by clinical characteristics and DST results. Rapid molecular testing would not sufficiently identify drug resistance in this population. Eligibility rates were not significantly reduced by prior TB treatment.

## Background

India is home to 27% of the world’s 10.4 million annual tuberculosis (TB) cases, making it the country with the highest TB burden in the world. [1, 2] India also has the world’s largest burden of multidrug resistant tuberculosis (“MDR-TB”, TB resistant to both isoniazid and rifampin), with an estimated 130,000 incident cases in 2016. [3, 4] Mumbai, a metropolis with ~ 1.5% of India’s population is particularly hard-hit. [5] While Mumbai accounts for ~ 2–3% of India’s TB burden, 12–13% of India’s MDR-TB patients are diagnosed in Mumbai, where TB incidence reaches 667 per 100,000 person-years. [6] Incomplete or improper treatment selects for resistant strains that can persist for years after selective drug pressure is withdrawn. [7, 8] The result is continued transmission of MDR-TB strains from person-to-person, including to those without prior TB treatment, as for 70% of extensively drug resistant (XDR) TB in South Africa. [9]

Drug resistance significantly impacts treatment outcomes. In India, treatment for drug-susceptible TB cures 84% of patients, while MDR-TB treatment succeeds for only 46%. [4] To improve treatment success, WHO has recommended that MDR-TB patients be considered for a short, intensive treatment regimen (“Short Course”) if they have pulmonary disease, < 1 month of MDR-TB treatment, are not pregnant, and are unlikely to have additional drug resistance. This consists of 4–6 months of a fluoroquinolone, kanamycin, prothionamide, high-dose isoniazid, clofazimine, pyrazinamide, and ethambutol, followed by 5 months of the fluoroquinolone, clofazimine, pyrazinamide, and ethambutol. [2, 3] Because of limited global drug susceptibility testing (DST) capacity, WHO does not require DST for 2nd-line drugs before starting Short Course. Instead, it allows treatment history and local epidemiology to guide eligibility without defining specific thresholds of local resistance above which Short Course should be avoided. MDR-TB in Mumbai is known to have complex resistance profiles including high rates of fluoroquinolone resistance and totally drug resistant tuberculosis (TDR-TB). [10–12] In order identify the proportion of such patients who are eligible for Short Course therapy by clinical and DST criteria, we reviewed clinical and laboratory data from an MDR-TB cohort in Mumbai..

## Methods

### Setting

The P.D. Hinduja National Hospital and Medical Research Centre (Hinduja Hospital) is a private, tertiary care hospital in Mumbai, India with an outpatient chest clinic and microbiology laboratory uniquely specialized for MDR-TB. The clinic sees ~ 3000 adults each year with a weekly free clinic. The laboratory processes > 32,000 TB samples annually and is accredited by the College of American Pathologists and the National Accreditation Board for Testing and Calibration Laboratories. Phenotypic testing at Hinduja Hospital includes 14 drug DST (isoniazid, rifampin, pyrazinamide, ethambutol, streptomycin, ofloxacin, moxifloxacin, amikacin, kanamycin, capreomycin, ethionamide, clofazimine, PAS, and linezolid). The lab also performs Xpert MTB/RIF (“Xpert”), line probe assays (“LPA”), and pyrosequencing. Phenotypic DST is performed in mycobacteria growth indicator tubes (MGIT) with the following concentrations: rifampin (1 μg/mL), ofloxacin (2 μg/mL), moxifloxacin (0.5 μg/mL and 2.0 μg/mL), kanamycin (2.5 μg/mL), amikacin (1 μg/mL), capreomycin (2.5 μg/mL), ethambutol (5 μg/mL), pyrazinamide (1 μg/mL at acidic pH), and clofazimine (1 μg/mL). [13, 14]

### Study sample and variables

Between October 20, 2015 and October 15, 2018, all MDR-TB patients presenting to the outpatient chest clinic were recruited for a prospective observational cohort. Participants were approached by a study clinician and provided informed consent for abstraction of their medical records onto paper data collection forms. Variables collected included demographic information (age, sex, occupation, smoking history, whether the participant left work for TB); TB presentation history (diagnosis in public or private sector, height, weight, and site of TB defined as pulmonary, extrapulmonary, or both); TB treatment history (names, doses, and dates of prior medications taken), the presence of prior TB episodes defined as discrete periods of illness treated ≥2 years before the current episode, city and state of prior TB treatment, laboratory and imaging studies (glycosylated hemoglobin, blood glucose, HIV test results, percentage of lung affected, and presence of a cavity on chest radiography), and symptoms during treatment. Diabetes was defined by a documented glycosylated hemoglobin ≥6.5 or two fasting glucose levels ≥125. Participants were followed at each subsequent unscheduled visit to assess treatment-associated side effects. Due to the observational nature of this cohort, not every participant had full records available for review. As a result, rates are presented using the number of participants with complete data available in the denominator.

### Statistical analysis

Data were collected on paper forms, entered in Microsoft Access (Office Professional 365, Microsoft Corporation, Redmond, Washington), and analyzed in R (version 3.3.2, R Core Team, Vienna, Austria). Records were reviewed for date of first MDR-TB-active drug (defined as a fluoroquinolone, amikacin, kanamycin, capreomycin, ethionamide, prothionamide, cycloserine, clofazimine, linezolid, or PAS). Time from first prescription of MDR-TB-active drug to first clinic visit was calculated to determine if participants had received < 1 month of MDR-TB treatment upon enrollment. DST profiles by phenotypic (MGIT) or molecular (Xpert or LPA) tests were abstracted from medical records and stratified by Short Course eligibility criteria. [15] Molecular and phenotypic DST results were considered equivalent and combined into a single variable. When multiple tests were available, each participant’s results were summarized as the most resistant available. Resistance was summarized for any fluoroquinolone (ofloxacin, levofloxacin, or moxifloxacin) or 2nd-line injectable (amikacin, capreomycin, or kanamycin). While all participants had confirmed resistance to both isoniazid and rifampin, data were compiled to represent the impact of DST performed for rifampin alone as would be tested by Xpert; by an LPA incorporating rifampin, a fluoroquinolone, and a 2nd-line injectable (“2nd-Line LPA”); by testing rifampin, a fluoroquinolone, a 2nd-line injectable, pyrazinamide, and ethambutol; and by rifampin, a fluoroquinolone, a 2nd-line injectable, pyrazinamide, ethambutol, ethionamide, and clofazimine.

To determine the impact of prior treatment on eligibility, resistance rates were calculated for participants with and without prior MDR-TB-active treatment. Differences in proportion of participants were evaluated by χ^2^ tests. Charts were reviewed for previously treated participants to identify location of prior TB treatment, which was classified as Mumbai, elsewhere in Maharashtra, or another state in India.

### Ethics approval and consent to participate

All study participants provided written informed consent for this study. Participants < 18 years of age had written consent provided by legal guardians and provided assent for study participation. This study was approved by the institutional review boards of Hinduja Hospital and Johns Hopkins University School of Medicine.

## Results

A total of 559 participants were enrolled during the study period. Participants had a median age of 29.8 years (interquartile range (IQR), 22–34 years) and a female predominance (349 participants, 62.4%, Table 1). Median body mass index at enrollment was 19.7 (IQR 16.7–23.0). Diabetes was confirmed in 36 participants. Only 39 participants reported ever smoking, and HIV was rare (2 participants, Table 1). Household exposure data were available for 496 participants, of whom 90 reported household contacts with MDR-TB (18.1%). Most participants were diagnosed in the private sector (44 of 525 participants with complete data, 84.6%) with an average of 2.2 medical providers before enrollment (IQR 1–3). Out of 556 participants with treatment records available, nearly half of participants had received MDR-TB treatment prior to the week of study enrollment (267 participants, 48.0%, Table 1), with a median time from diagnosis to enrollment of 2.6 months (IQR 0.7–10.5 months). Compared to those without prior MDR-TB treatment, treatment before enrollment was associated with higher rates of diagnosis in the private sector, leaving work or school due to TB, diabetes, and smear positivity (Table 1). No other significant differences were found between participants according to previous MDR-TB treatment.

**Table 1:** Demographic and Clinical Characteristics of 559 Participants with MDR-TB Treated at Hinduja Hospital, by Prior MDR-TB Treatment

	Prior MDR-TB Treatment, *N* = 267	No Prior MDR-TB Treatment, *N* = 289	All Participants, *N* = 559	Χ^2^ Test
	#/# with Complete Data^a^ (%)	#/# with Complete Data^a^ (%)	#/# with Complete Data^a^ (%)	*p*-value
Female	163 / 267 (61.0)	184 / 289 (63.7)	349 / 559 (62.4)	0.583
Diagnosed in Private Sector	227 / 254 (89.4)	215 / 269 (79.9)	444 / 525 (84.6)	0.004
Current Student	66 / 267 (24.7)	67 / 289 (23.2)	133 / 559 (23.8)	0.746
Health Care Worker	13 / 267 (4.9)	19 / 289 (6.6)	33 / 559 (5.9)	0.889
Left Work or School Due to TB	107 / 267 (40.1)	140 / 289 (48.4)	247 / 559 (44.2)	0.026
Current or Former Smoker	11 / 214 (5.1)	28 / 268 (10.4)	39 / 485 (8.0)	0.051
Household Contact with MDR-TB	35 / 224 (15.6)	55 / 269 (20.4)	90 / 496 (18.1)	0.207
Pulmonary TB Only	192 / 267 (71.9)	221 / 289 (76.5)	415 / 559 (74.2)	0.168
Cavitary Lesions on X-ray	118 / 227 (52.0)	146 / 241 (60.6)	266 / 470 (56.6)	0.075
HIV Positive	1 / 138 (0.7)	1 / 163 (0.6)	2 / 301 (0.7)	1.000
Diabetic	18 / 99 (18.2)	18 / 49 (36.7)	36 / 148 (24.3)	0.023
Smear Positive	163 / 267 (61.0)	207 / 289 (71.6)	372 / 559 (66.5)	0.011
Culture Positive	227 / 253 (89.7)	254 / 277 (91.7)	483 / 532 (90.8)	0.527

^a^Due to the observational nature of this cohort study, complete records were not available for all study participants. Denominators are adjusted in each field to reflect data completion among study participants

**Fig. 1:**
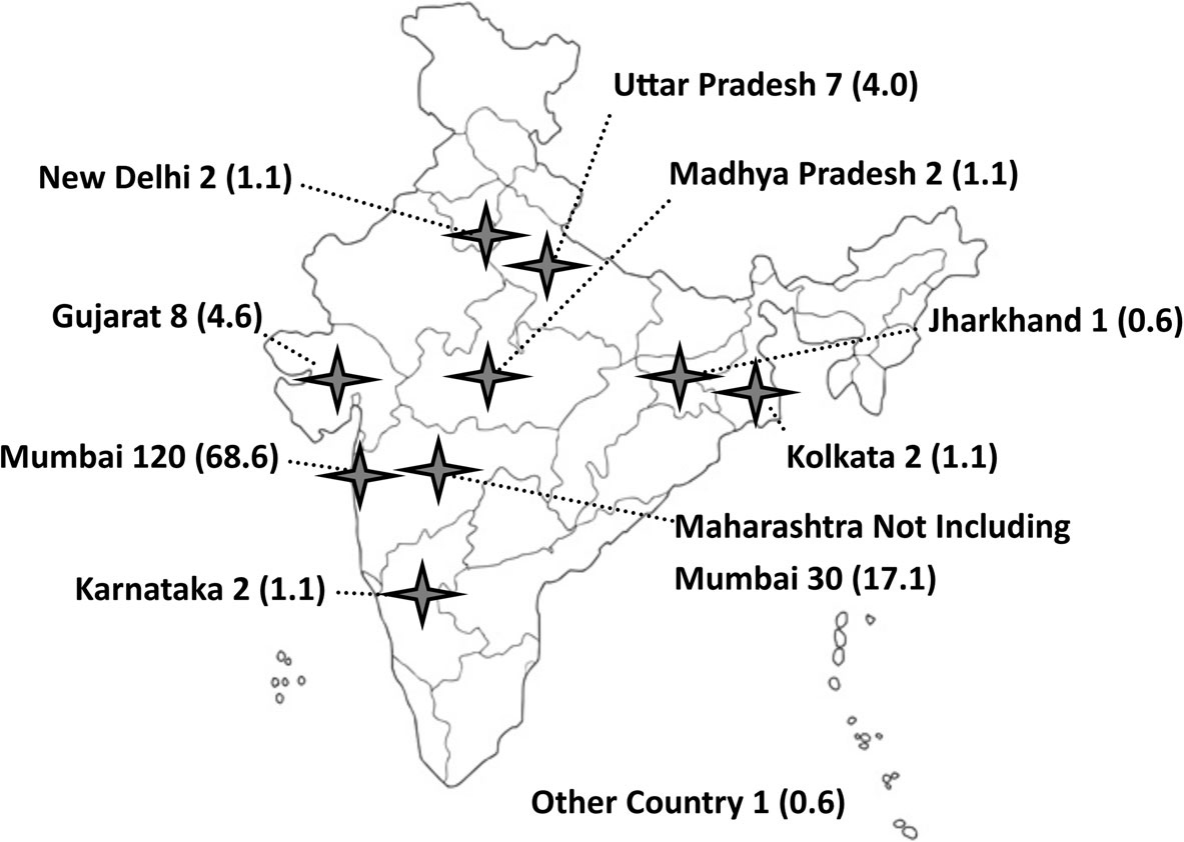
Prior TB Treatment Locations in India of 102 Participants with MDR-TB Treated in Mumbai, N (%). Participants with MDR-TB in this cohort received prior TB treatment throughout India, not only in Mumbai. The authors have edited an original image obtained from www.shutterstock.com (ID: 225879508)

**Fig. 2:**
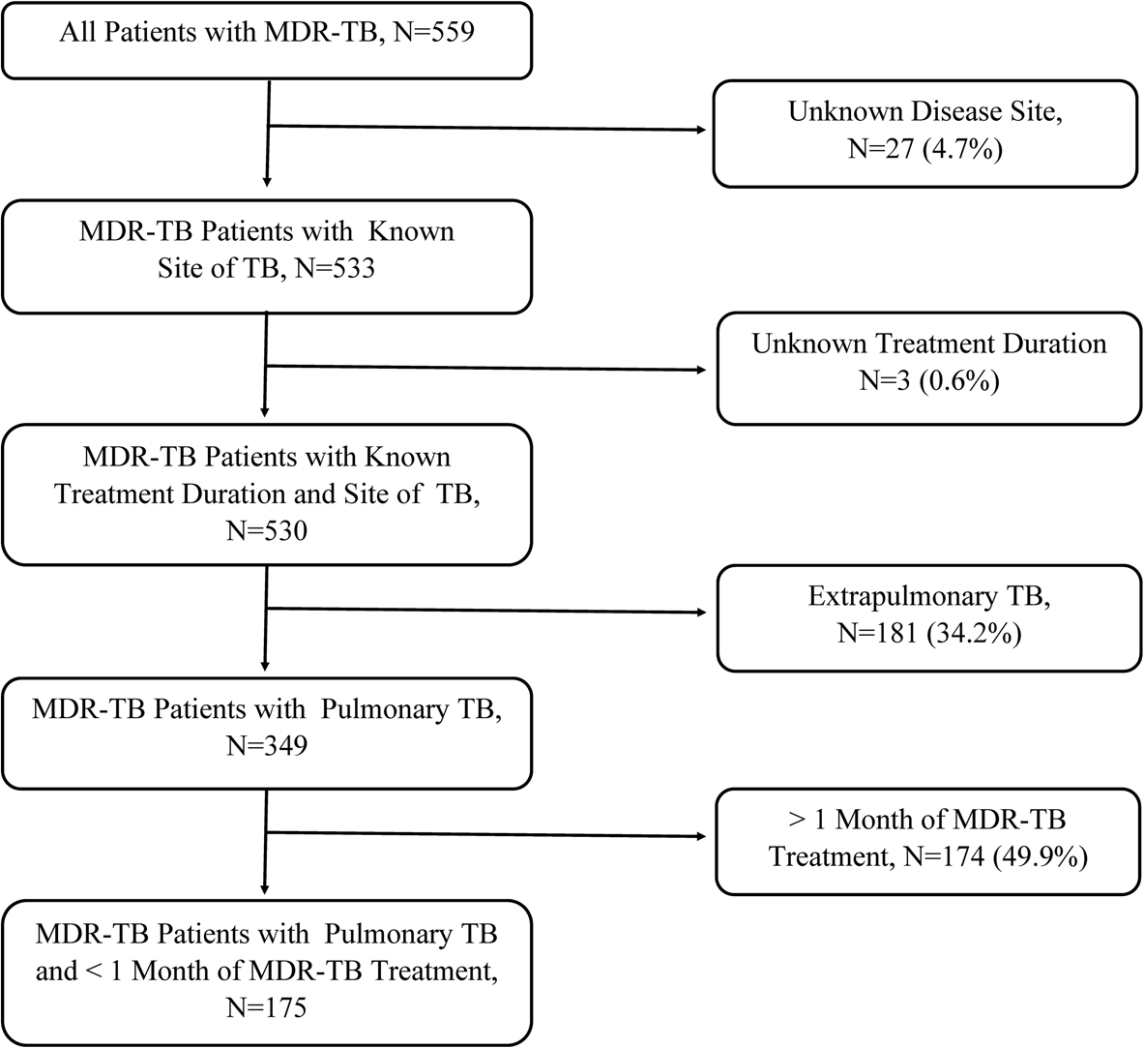
Eligibility for Short Course Therapy by Clinical Criteria. Only 175 out of 530 participants with MDR-TB and full clinical data available met clinical criteria for Short Course treatment (33.0%)

Many previously treated participants received > 1 month of MDR-TB treatment prior to enrollment (258 of 556 participants with known treatment duration, 46.4%). Of these, 66 participants (11.9% of those with prior treatment data) had been treated with the combination of 1st-line drugs, streptomycin, and a fluoroquinolone together. Prior episodes of TB were common, with 137 participants (24.6%) reporting TB episodes ≥2 years prior to their current illness. Location of prior treatment was reported by 175 participants, with 120 reporting prior treatment in Mumbai (68.6%) and 55 of those 175 participants reporting treatment elsewhere in India (31.4%, Fig. 1). Complete clinical data for eligibility screening was available for 530 participants, of whom only 175 were eligible for Short Course by clinical criteria alone (33.0%, Fig. 2).

Among the entire cohort of 559 participants, 2184 microbiological samples were tested for TB (1955 by smear microscopy, 593 by Xpert, 57 by LPA, and 1578 by phenotypic DST). If only rifampin resistance testing were required (as with Xpert), 559 participants would be considered eligible for Short Course treatment (Table 2). If DST were performed for rifampin, fluoroquinolones, and 2nd-line injectables (as with a 2nd-line LPA), only 118 of the 466 participants with such test results would remained eligible (25.3%). Performing DST for all Short Course drugs reduced eligibility further to only 14 of 407 participants with complete DST results available (3.4%, Table 2).

**Table 2:** Eligibility for Short Course Regimen by Clinical Criteria and DST Performed. a. Participants with MDR-TB Eligible for Short Course Treatment, by DST Performed

Resistance Status	Xpert Only, *N* = 559	2nd-Line LPA Only,^a^ *N* = 466	Testing R, FQ, INJ, Z, and E, *N* = 432^b^	Testing R, FQ, INJ, Z, E, Eto, and Cfz, *N* = 407^b^
	# Eligible or Resistant / # Tested (%)^c^	# Eligible or Resistant / # Tested (%)^c^	# Eligible or Resistant / # Tested (%)^c^	# Eligible or Resistant / # Tested (%)^c^
Eligible for Short Course	559 / 559 (100)	118 / 466 (25.3)	16 / 432 (3.7)	14 / 407 (3.4)
Resistant to FQ or INJ	N/A	348 / 466 (74.7)	320 / 432 (74.1)	304 / 407 (74.7)
Resistant to Z or E	N/A	N/A	408 / 432 (94.4)	386 / 407 (94.8)
Resistant to FQ, INJ, Z, or E	N/A	N/A	416 / 432 (96.3)	391 / 407 (96.1)
Resistant to FQ, INJ, Z, E, Eto, or Cfz	N/A	N/A	N/A	393 / 407 (96.6)

^a^2^nd^-line LPA tests for susceptibility to isoniazid, rifampin, fluoroquinolones, and 2nd-line injectables (amikacin, capreomycin, or kanamycin)

^b^*R* rifampin, *FQ* fluoroquinolone, *INJ* 2nd-line injectable, *Z* pyrazinamide, *E* ethambutol, *Eto* ethionamide, *Cfz* clofazimine

^c^Percentages reported reflect number of participants with resistance or susceptibility divided by number of participants who completed susceptibility testing for each drug. As a result, the number of participants in the denominator is not the same in all rows

Of 267 MDR-TB-treatment naive participants, 217 completed DST for fluoroquinolones and 2nd-line injectable drugs. Only 67 of those 217 participants (30.9%) remained eligible after DST for rifampin, fluoroquinolones, and 2nd-line injectables. Performing DST for all drugs in the regimen identified only 10 MDR-TB treatment naïve participants to be eligible for Short Course (5.0% of the 200 with DST results for all Short Course drugs, Table 3).

**Table 3:** Eligibility for Short Course Regimen by Clinical Criteria and DST Performed. b. Participants with MDR-TB Eligible for Short Course Treatment Who Had Not Previously Received MDR-TB-Active Treatment, by DST Performed

Resistance Status	Xpert Only, *N* = 267	2nd-Line LPA Only,^a^ *N* = 217	Testing R, FQ, INJ, Z, and E, *N* = 210^b^	Testing R, FQ, INJ, Z, E, Eto, and Cfz, *N* = 200^b^
	# Eligible or Resistant / # Tested (%)^c^	# Eligible or Resistant / # Tested (%)^c^	# Eligible or Resistant / # Tested (%)^c^	# Eligible or Resistant / # Tested (%)^c^
Eligible for Short Course	267 / 267 (100)	67 / 217 (30.9)	11 / 210 (5.2)	10 / 200 (5.0)
Resistant to FQ or INJ	N/A	150 / 217 (69.1)	143 / 210 (68.1)	135 / 200 (67.5)
Resistant to Z or E	N/A	N/A	194 / 210 (92.4)	185 / 200 (92.5)
Resistant to FQ, INJ, Z, or E	N/A	N/A	199 / 210 (94.8)	189 / 200 (94.5)
Resistant to FQ, INJ, Z, E, Eto, or Cfz	N/A	N/A	N/A	190 / 200 (95.0)

^a^2^nd^-line LPA tests for susceptibility to isoniazid, rifampin, fluoroquinolones, and 2nd-line injectables (amikacin, capreomycin, or kanamycin)

^b^*R* rifampin, *FQ* fluoroquinolone, *INJ* 2nd-line injectable, *Z* pyrazinamide, *E* ethambutol, *Eto* ethionamide, *Cfz* clofazimine

^c^Percentages reported reflect number of participants with resistance or susceptibility divided by number of participants who completed susceptibility testing for each drug. As a result, the number of participants in the denominator is not the same in all rows

Only 175 of the 530 participants with complete clinical data (33.0%) remained Short Course-eligible by the combination of clinical criteria (pulmonary disease and < 1 month of MDR-TB active drugs) and rifampin resistance (Fig. 2, Table 4). Among these, 46 participants remained eligible after DST for rifampin, fluoroquinolones, and 2nd-line injectables (31.5% of 146 participants with DST results), and 8 remained eligible after complete DST (5.9% of 136 participants with DST results for all Short Course drugs). The addition of ethionamide and clofazimine DST to testing for rifampin, fluoroquinolones, 2nd-line injectables, pyrazinamide, and ethambutol excluded only one additional participant (Tables 2, 3 and 4).

**Table 4:** Eligibility for Short Course Regimen by Clinical Criteria and DST Performed. c. Participants with Pulmonary MDR-TB Eligible for Short Course Treatment with < 1 Month of MDR-TB Treatment, by DST Performed

Resistance Status	Xpert Only, *N* = 175	2nd-Line LPA Only,^a^ *N* = 146	Testing R, FQ, INJ, Z, and E, *N* = 140^b^	Testing R, FQ, INJ, Z, E, Eto, and Cfz, *N* = 136^b^
	# Eligible or Resistant / # Tested (%)^c^	# Eligible or Resistant / # Tested (%)^c^	# Eligible or Resistant / # Tested (%)^c^	# Eligible or Resistant / # Tested (%)^c^
Eligible for Short Course	175 / 175 (100)	46 / 146 (31.5)	9 / 140 (6.4)	8 / 136 (5.9)
Resistant to FQ or INJ	N/A	100 / 146 (68.5)	95 / 140 (67.9)	93 / 136 (68.4)
Resistant to Z or E	N/A	N/A	128 / 140 (91.4)	124 / 136 (91.2)
Resistant to FQ, INJ, Z, or E	N/A	N/A	131 / 140 (93.6)	127 / 136 (93.4)
Resistant to FQ, INJ, Z, E, Eto, or Cfz	N/A	N/A	N/A	128 / 136 (94.1)

^a^2^nd^-line LPA tests for susceptibility to isoniazid, rifampin, fluoroquinolones, and 2nd-line injectables (amikacin, capreomycin, or kanamycin)

^b^*R* rifampin, *FQ* fluoroquinolone, *INJ* 2nd-line injectable, *Z* pyrazinamide, *E* ethambutol, *Eto* ethionamide, *Cfz* clofazimine

^c^Percentages reported reflect number of participants with resistance or susceptibility divided by number of participants who completed susceptibility testing for each drug. As a result, the number of participants in the denominator is not the same in all rows

Prior treatment with MDR-TB-active drugs was associated with higher rates of resistance to ethambutol, fluoroquinolones, aminoglycosides, and PAS (Table 5). There was no statistically significant difference in resistance rates to any drugs tested when participants were stratified by prior treatment with first line drugs (isoniazid, pyrazinamide, or ethambutol, data not shown).

**Table 5:** Drug Resistance Among 559 Participants with MDR-TB, by Prior Drug of interest and MDR-TB Treatment Status

Drug	Received Any Prior MDR-TB Treatment, *N* = 267^a^	Received No Prior MDR-TB Treatment, *N* = 289^a^	All Participants, *N* = 559^a^	Χ^2^ Test
	# Resistant / # Tested (%)^b^	# Resistant / # Tested (%)^b^	# Resistant / # Tested (%)^b^	*p*-value
Isoniazid	246 / 248 (99.2)	217 / 219 (99.1)	465 / 469 (99.1)	1.000
Rifampin	289 / 289 (100)	267 / 267 (100)	559 / 559 (100)	1.000
Pyrazinamide	185 / 228 (81.1)	176 / 213 (82.6)	363 / 443 (81.9)	0.778
Ethambutol	212 / 237 (89.5)	178 / 219 (81.3)	392 / 458 (85.6)	0.019
Streptomycin	167 / 177 (94.4)	91 / 100 (91.0)	260 / 279 (93.2)	0.417
Ofloxacin	183 / 233 (78.5)	141 / 211 (66.8)	326 / 446 (73.1)	0.008
Moxifloxacin (low)	157 / 229 (68.6)	122 / 208 (58.7)	281 / 439 (64.0)	0.040
Moxifloxacin (high)	34 / 145 (23.4)	30 / 178 (16.9)	65 / 324 (20.1)	0.181
Kanamycin	73 / 244 (29.9)	35 / 217 (16.1)	110 / 463 (23.8)	< 0.001
Amikacin	51 / 225 (22.7)	23 / 206 (11.2)	76 / 433 (17.6)	0.002
Capreomycin	48 / 235 (20.4)	22 / 212 (10.4)	72 / 449 (16.0)	0.005
Ethionamide	150 / 231 (64.9)	122 / 218 (56.0)	274 / 451 (60.8)	0.065
PAS	72 / 231 (31.2)	28 / 217 (12.9)	101 / 450 (22.4)	< 0.001
Clofazimine	8 / 219 (3.7)	3 / 204 (1.5)	11 / 425 (2.6)	0.270
Linezolid	13 / 136 (9.6)	6 / 171 (3.5)	20 / 308 (6.5)	0.052

^a^All Participants column includes 3 participants for whom the history of prior therapy was not documented

^b^Percentages reported reflect number of participants with resistance divided by number of participants who completed susceptibility testing for each drug. As a result, the number of participants in the denominator is not the same in all rows

## Discussion

This study evaluated clinical and laboratory features of 559 participants with MDR-TB in Mumbai to determine Short Course MDR-TB treatment eligibility. The cohort was young and female-predominant with low prevalence of smoking, diabetes, and HIV. Most participants at this large referral center were diagnosed within 3 months of enrollment, though several participants reported previous TB treatment, often in other Indian states (Fig. 1). If all participants had DST performed for fluoroquinolone and 2nd-line injectable drugs (as in a 2nd-line LPA like the Hain Genotype MTBDRplus), < 1/3rd would remain eligible for Short Course treatment. The addition of DST to pyrazinamide, ethambutol, ethionamide, and clofazimine found that < 1 in 20 participants with MDR-TB would be expected to benefit from the Short Course regimen. Similar rates of eligibility were found among those with prior MDR-TB treatment and those meeting both DST and clinical criteria. While many participants had previously received treatment, resistance to second line drugs was common regardless of prior TB treatment, reflecting the high rates of resistance among infecting strains, rather than treatment failure.

Mumbai, a city where complex drug resistance is common, represents a challenge for the WHO-recommended Short Course regimen. With rising MDR-TB rates and a renewed interest in transmitted resistance, it is more important than ever to identify successful treatment strategies. On an individual level, pyrazinamide, ethambutol, and ethionamide each substantially improve the odds of successful treatment. [16, 17] At a national level, optimizing MDR-TB treatment could reduce incidence and mortality in India by 32 and 30%, respectively. [18] For both the individual and for society, it is incredibly important to implement testing and treatment strategies with high likelihoods of success.

The WHO Short Course strategy is supported by good observational data. [15] Unfortunately, our analysis of 559 study participants with MDR-TB recruited over 3 years suggests that this regimen has limited application in our setting. Clinical criteria alone limited eligibility to 33% of MDR-TB participants, and full DST data suggest that < 5% would benefit from Short Course treatment. These findings were independent of either prior treatment regimens or site of infection (pulmonary or extrapulmonary). Though prior treatment with MDR-TB-active drugs demonstrated a statistically significant impact on rates of resistance to specific drugs, both previously treated and treatment-naïve participants faced high rates of drug resistance (Table 5). Many participants were treated before enrollment, but that treatment did not always follow national guidelines, evidenced by the frequent simultaneous use of first line therapy, streptomycin, and a fluoroquinolone (66 of 556 previously treated participants). This is consistent with the increase in quinolone and injectable drug resistance identified in Table 5. Though this study did not evaluate transmission patterns, it is possible that these resistance profiles reflect circulating resistance in Mumbai, rather than the treatment received before enrollment. Several participants had contacts with MDR-TB, suggesting transmission of these strains at home or in the workplace, which may be different from the drug susceptible strains circulating in the community.

Mumbai is known to have high rates of complex drug resistance including fluoroquinolone resistance, XDR-TB, and TDR-TB, but similar resistance rates are not reported elsewhere in India. [10–12] The average global rate of fluoroquinolone resistance among MDR-TB isolates is 20%, and XDR-TB represents only 6.2% of global MDR-TB. [1, 2] Part of the difference between our resistance rates and those reported elsewhere may be explained by infrequent phenotypic DST for pyrazinamide, ethambutol, ethionamide, and clofazimine outside of major referral laboratories. That said, the high proportion of participants in this cohort receiving prior treatment in other Indian states (55 participants, 31.4% of 175 previously treated participants), suggests that additional DST is worth considering prior to implementing Short Course elsewhere in India.

This study had several limitations. First, both the clinic and lab evaluate and manage complex TB drug resistance, likely resulting in a recruitment bias. This analysis stratified participants by prior treatment with MDR-TB active drugs but could not completely resolve this bias. Second, the fact that most participants are from Mumbai limits the generalizability of the findings. Additional research is needed to evaluate resistance to ethambutol, pyrazinamide, ethionamide, and clofazimine in other parts of India to confirm whether our findings match those of the general population elsewhere. It is important to note, however, that Mumbai reports a disproportionately high number of India’s MDR-TB patients and therefore data from Mumbai remain highly relevant to the global MDR-TB epidemic. [6] Third, we recruited from a chest clinic rather than an HIV clinic, so the rate of HIV infection in this cohort approximates that of India’s national prevalence of HIV among adults (0.7 and 0.26%, respectively). [19] This is not a novel finding, as 89% of new TB cases worldwide occur in HIV-negative people, [20] but the low HIV prevalence in our data may limit generalizability of data from this cohort to high-burden settings for HIV. Fourth, phenotypic testing of pyrazinamide and ethambutol is technically difficult, which limits the expectation that the DST performed at our intermediate level lab could be easily replicated in all testing centers. Finally, the Short Course regimen has been tested as a combination of drugs, rather than as individual drugs used one-by-one. [21, 22] It is not clear the extent to which resistance to any individual component drugs will directly impact treatment-associated outcomes. Likewise, the critical concentrations at which most TB drugs are tested were not determined with respect to clinical outcomes, so the correlation between phenotypic resistance to any given drug and successful treatment is uncertain, reflected by the demonstrated good outcomes of Short Course treatment despite pyrazinamide resistance. [15, 23, 24] Pooled analyses have attempted to address this problem, but until further prospective studies are performed to evaluate the individual drugs in the Short Course regimen, this will remain a limitation of our data. [16, 17]

Treatment decisions for MDR-TB are complex. When DST is not available, guidelines suggest that local epidemiology guide individual treatment decisions. No specific population resistance threshold is offered to determine whether a drug should be used empirically. Data from other countries have found that ~ 50% of patients from Brazil and Pakistan, ~ 30% of patients in Singapore, and ~ 10% of European patients would be ineligible for Short Course treatment. [25–29] In higher burden areas like Eastern Europe, these rates are even lower (4.2%). [30] The most recent Indian program guidelines recommend that Short Course be employed following rifampin resistance testing. [31] If no further testing were performed, our data show that < 5% of those patients would receive treatment to which their isolates are susceptible. These data suggest that rates of resistance to the drugs in the Short Course regimen among MDR-TB patients in Mumbai are too high for empiric therapy to work here. Previous publications from this hospital suggest that the Category IV treatment regimen employs drugs with to which 66.5% of MDR-TB patients are resistant. [32] Similarly, this study confirms that the Short Course regimen would rely on drugs to which 96.8% of MDR-TB patients in our clinic are resistant.

## Conclusions

Though it may benefit the minority of participants for whom DST confirms susceptibility to component drugs, the Short Course regimen appears to be a suboptimal choice for empiric therapy in our setting. This applies equally if only an Xpert or LPA are performed for DST. Given the low rates of additional resistance identified only by testing ethionamide and clofazimine, it is possible that DST for those drugs is less important than incorporating pyrazinamide and ethambutol testing. At least for the time being, phenotypic DST must remain a priority in evaluating MDR-TB patients in areas with high rates of complex drug resistance like Mumbai, India.

## Abbreviations

2nd-Line Injectables: Amikacin, capreomycin, or kanamycin
2nd–Line LPA: A line probe assay incorporating rifampin, a fluoroquinolone, and a second-line injectable drug (amikacin, kanamycin, or capreomycin)
Cfz: Clofazimine
DST: Drug susceptibility testing
E: Ethambutol
Eto: Ethionamide
FQ: Fluoroquinolone
Hinduja Hospital: P.D. Hinduja National Hospital and Medical Research Centre
INJ: Amikacin, capreomycin, or kanamycin
IQR: Interquartile range
LPA: Line probe assay
MDR-TB: Multidrug resistant tuberculosis, defined as tuberculosis resistant to both isoniazid and rifampin
MGIT: Mycobacteria growth indicator tube
R: Rifampin
Short Course: A short, intensive treatment regimen for tuberculosis consisting of 4–6 months of a fluoroquinolone, kanamycin, prothionamide, high-dose isoniazid, clofazimine, pyrazinamide, and ethambutol, followed by 5 months of the fluoroquinolone, clofazimine, pyrazinamide, and ethambutol
TB: Tuberculosis
TDR-TB: Totally drug resistant tuberculosis, defined as tuberculosis resistant to all antituberculosis drugs
XDR-TB: Extensively drug resistant tuberculosis, defined as tuberculosis resistant to isoniazid, rifampin, a fluoroquinolone, and a second-line injectable drug (amikacin, kanamycin, or capreomycin)
Xpert: Xpert MTB/RIF
Z: pyrazinamide
